# Transcranial Electrical Stimulation for Relief of Peripartum Mental Health Disorders in Women Undergoing Cesarean Section With Combined Spinal–Epidural Anesthesia: A Pilot Randomized Clinical Trial

**DOI:** 10.3389/fpsyt.2022.837774

**Published:** 2022-04-04

**Authors:** Qiu Zhao, Yuan Han, Xiao-Yi Hu, Song Zhang, Long Zhang, Jun Wang, Qian-Qian Zhang, Ming-Shu Tao, Jia-xing Fang, Jie Yang, Rong-Guang Liu, Xun Sun, Jian Zhou, Xiang Li, Mannan-Abdul, Hongxing Zhang, He Liu, Jun-Li Cao

**Affiliations:** ^1^Department of Anesthesiology, The Affiliated Hospital of Xuzhou Medical University, Xuzhou, China; ^2^Jiangsu Province Key Laboratory of Anesthesiology and NMPA Key Laboratory for Research and Evaluation of Narcotic and Psychotropic Drugs, Xuzhou Medical University, Xuzhou, China; ^3^Department of Anesthesiology, Eye & ENT Hospital of Fudan University, Shanghai, China; ^4^Department of Anesthesiology, Renji Hospital School of Medicine Shanghai Jiao Tong University, Shanghai, China; ^5^Insititute of Nervous System Diseases, Xuzhou Medical University, Xuzhou, China; ^6^Department of Anesthesiology, The Affiliated Huzhou Hospital, Zhejiang University School of Medicine, Huzhou Central Hospital, Huzhou, China

**Keywords:** transcranial electrical stimulation, mental health disorders, cesarean section, peripartum anxiety, peripartum depression

## Abstract

**Objective:**

This study aimed to explore transcranial electrical stimulation (tES) to relieve peripartum anxiety and depressive symptoms in women undergoing cesarean section with combined spinal–epidural anesthesia.

**Methods:**

This double-blind, randomized, sham-controlled trial was conducted in the Affiliated Hospital of Xuzhou Medical University from March 2021 and May 2021. One hundred and forty-eight full-term parturients giving birth by elective cesarean section were selected, and 126 were included in the intent-to-treat analysis. Parturients were provided standardized anesthesia and randomized to the active-tES (a-tES) group and sham-tES group. Parturients and outcome assessors were blinded to treatment allocation. The primary outcome was the changes in peripartum mental health disorders, including anxiety, assessed by the Pregnancy-Related Anxiety Questionnaire-Revised 2 (PRAQ-R2). Secondary outcomes included peripartum depressive symptoms, assessed by the Edinburgh Postnatal Depression Scale (EPDS), maternal satisfaction, fatigue level, sleep quality index, and pain score during and after operation. Data were collected before entering the operating room (T0), between post-anesthesia and pre-surgery (T1), before leaving the operating room (T2), and at 24 h post-surgery (T3).

**Results:**

One hundred and twenty-six eligible parturients were enrolled in the two groups: a-tES group (N = 62) and sham-tES group (N = 64). Treatment with tES resulted in significantly lower scores of anxiety compared with sham-tES (T2: *P* < 0.001; T3: *P* = 0.001). Moreover, the a-tES groups showed a significant reduction in depression scores (T2: *P* = 0.003; T3: *P* = 0.032).

**Conclusion:**

In this randomized pilot study, tES treatment is efficacious in alleviating peripartum anxiety and depressive symptoms in women undergoing cesarean section and has been demonstrated to be a novel strategy for improving peripartum mental health disorders.

**Clinical Trial Registration:**

[www.chictr.org.cn], identifier [ChiCTR2000040963].

## Introduction

Peripartum anxiety and/or depression is a common mental health disorder during pregnancy (1, 2). The overall prevalence during pregnancy is 15.2% for any anxiety disorder and 22.9% for anxiety symptoms (3); furthermore, peripartum depression has been nominated as a common complication of pregnancy and affects one in every seven women (4). Sixty percent of women with peripartum depression have preexisting comorbid psychiatric disorders, of which more than 80% are anxiety disorders (5). Peripartum mental health disorders can lead to poor maternal–infant physical health and negative birth outcomes (6–8). Studies have shown that pregnant women with anxiety and/or depression have experienced more nausea and vomiting, show instability in their professional behavior, and visit the obstetrician more frequently during pregnancy compared to pregnant women without anxiety and/or depression (9). Moreover, women with peripartum anxiety are more likely to experience preterm birth, have infants with lower than average birth weight (i.e., <2,500 g), and have infants with increased probability of being admitted to the NICU (10). Meta-analysis from 17 pooled studies showed that peripartum anxiety was significantly associated with preterm birth: 5 pooled studies showed a significant effect on spontaneous preterm birth, and 12 pooled studies showed lower infant birth weight (3). Moreover, if these disorders are being ignored or left untreated, they have adverse effects on women and their children, ranging from increased risk of poor adherence to medical care, exacerbation of medical conditions, loss of interpersonal and financial resources, smoking and drug addiction, suicide, and infanticide (11). Therefore, peripartum mental health disorders, including anxiety and/or depression, are associated with increased risks of maternal and infant mortality and morbidity and are recognized as a significant patient safety issue (12).

Peripartum anxiety and/or depression is often underdiagnosed and inadequately treated (5). The current recommended method for anxiety disorders in the general population is psychotherapy (13); however, its efficacy is not definitive. It has been documented that women who experience symptoms of anxiety and depression are commonly prescribed antidepressants. However, before becoming pregnant, they mostly abandon taking drugs because of the insecurities regarding the potential teratogenicity of the antidepressants (14, 15).

Considering the significant impact of peripartum mental health disorders on both the mother and the newly born child, it is imperative to explore effective therapeutic strategies. Non-pharmacological and non-invasive interventions are innovative approaches that can be a feasible strategy for the treatment of mental health disorders (16, 17). Research shows that psychiatric disorders might result from a maladaptive neuroplasticity of the prefrontal and limbic regions, with hypoactivation of the DLPFC (18–20) or abnormalities in amygdala processing (21, 22). tES is a non-invasive method of applying low-intensity electrical current to the DLPFC for treating neurological conditions and psychiatric disorders (23, 24). tES can be anticipated as a therapy for anxiety, pain, insomnia, depression, headache, fibromyalgia, and numerous affective disorders (25, 26). An early meta-analysis by Klawansky and colleagues (27) identified eight sham-controlled randomized trials for anxiety; the notable result arising from the meta-analysis was that the pooled result for the eight studies analyzing the treatment of anxiety with continuous scales was in favor of tES at a statistically significant level (effect size estimate = −0.5883; 95% confidence interval = −0.9503, −0.2262), and the result in favor of tES remained significant when they dropped the three studies that provided no convincing sensation in their sham protocol.

Although tES can be beneficial in relieving anxiety (28) and other psychiatric disorders in the general population, as far as we know, there are no randomized controlled trials to evaluate the effects of tES on peripartum mental health disorders. The main objective of this pilot study was to explore the novel strategy, tES, to relieve peripartum mental health disorders, including anxiety and depressive symptoms, in women undergoing cesarean section. The secondary aim was to evaluate the effects of the intervention on postpartum fatigue, maternal satisfaction, and Visual Analogue Scale (VAS) score during and after the operation.

## Materials and Methods

For this pilot randomized clinical trial, ethical approval was obtained from the Ethical Committee of the Affiliated Hospital of Xuzhou Medical University (ethics identifier XYFY2021-KL040-01, Chairperson Prof Tie Xu), Jiangsu, China, on 18 March 2021. The study was registered on the Chinese clinical trial registry^1^ with the identifier ChiCTR2000040963. All procedures performed in the study involving human participants were following the ethical standards of the institutional and/or national research committee, the 2013 Declaration of Helsinki, and the Consolidated Standards of Reporting Trials (CONSORT) reporting guideline (29). Written informed consent was obtained from all subjects participating, a legal surrogate, or the parents in this trial.

### Study Design and Settings

The study adopted a double-blind, a randomized sham-controlled clinical trial. From March 2021 to May 2021, parturients were recruited at the Affiliated Hospital of Xuzhou Medical University.

### Participants

One hundred and forty-eight full-term parturients giving birth by elective cesarean section were recruited at the Affiliated Hospital of Xuzhou Medical University. Inclusion criteria were (1) elective cesarean section, 38–42 weeks of gestational age, and good fetal heartbeat (120–160 bpm); (2) desire for combined spinal–epidural anesthesia; and (3) ASA class II (30). Exclusion criteria were (1) age younger than 18 years or older than 45 years; (2) ASA classes I, III, and IV; (3) eclampsia during pregnancy or cerebrovascular diseases; (4) experience with tES, forehead skin damage, or allergy; (5) intracorporeal implantation of electronic devices (e.g., pacemakers or other metal devices); (6) preexisting mental illness (but not anxiety disorders and depression) or history of psychotropic substance use within one month; and (7) inability to understand or refusal to sign informed consent.

### Randomization and Blinding

The principal investigators identified and enrolled patients. Using a permuted block method (31) (with a block size of 4 or 6) and an interactive voice–web response system, patients were randomly (1:1) assigned to receive a-tES or sham-tES. The follower and patients were blinded to treatment assignment. Group allocations were kept in opaque sealed envelopes sequentially numbered and disclosed by a health care practitioner not directly involved in the parturients’ clinical management and data collection. Each code was revealed just as the parturients entered the operating room to determine which stimulation methods would be prepared. All parturients used equipment, but for the sham control group, it would turn off automatically after 30 s, so the patients were blinded for grouping. The equipment was withdrawn immediately after the stimulation, so the follower was not aware of the grouping.

### Inventions

Standardized anesthesia was provided, including fasting for 6–8 h before anesthesia. Upon arrival into the operating room, the women were placed supine with the bed tilted 30° to the left. Standard monitoring was applied, consisting of electrocardiography (ECG), non-invasive blood pressure, and continuous pulse oximetry, and then the nurse would open the upper limb veins. All parturients were then placed in the left lateral position, and an anesthetist with at least 5 years’ experience performed combined spinal–epidural anesthetic using a needle-through-needle set at the estimated L2–L3 or L3–L4 vertebral interspace. Specifically, after skin disinfection and local skin infiltration with 2% lidocaine, an 18G Tuohy epidural needle was used to identify the epidural space, applying the loss of resistance method through the midline approach. A 27G Whitacre spinal needle with a pencil-point tip was passed through the Tuohy needle into the subarachnoid space. Entry into the subarachnoid space was confirmed by free cerebrospinal fluid outflow. Subarachnoid medication consisted of 0.75% bupivacaine hydrochloride 1.0–1.5 ml. The spinal needle was then withdrawn, and an epidural catheter was threaded into the epidural space. The epidural catheter was gently aspirated and checked for the absence of cerebrospinal fluid, and the epidural needle was removed with an epidural catheter inserted 3–5 cm into the epidural space in a cephalad direction.

After the intrathecal injection, parturients were turned supine; after maternal vital signs turned stable and during the time before surgery begins, the study staff again assessed the Pregnancy-Related Anxiety Questionnaire-Revised 2 (PRAQ-R2) and Edinburgh Postnatal Depression Scale (EPDS) scales, which would be completed within 5–10 min. Assessment of sensory block was done in each dermatomal level bilaterally for loss to cold sensation, 5 ml 2% lidocaine would be administrated for supplement of analgesia by epidural catheter if necessary, and the operation was to start only when the sensory block level was not below T6. Then gel electrodes were immediately placed on the frontal skull, and the tES equipment (GY168A) delivered a direct current of 2 mA (2 mA/5 cm × 2 cm = 0.2 mA/cm^2^; maximum energy output: 2 mA; pulse width: 250 μs; frequency: 120 Hz; duration: 20 min) to parturients in the a-tES group. For the sham-tES group, electrodes were placed on the same place, and sham stimulation (maximum energy output: 2 mA; pulse width: 250 μs; frequency: 120 Hz; duration: 30 s) was given to parturients. Following previously established methods of clinical studies of brain stimulation, the current was turned off automatically after 30 s of stimulation (32, 33). These methods provide the same initial sensory feelings of tES conditions, specifically, itching and tingling feelings on the scalp for the first few seconds of tES (32, 33). Both groups reported experiencing the same sensation during the 30 s period, and no participants described any differences between the conditions. Assessment of sensory block was done in each dermatomal level bilaterally for loss to cold sensation, and the operation was to start only when the sensory block level was not below T6. The anesthesiologists decided whether to add supplemental IV fluids and intraoperative analgesia.

### Data Collection

#### General Information

After admission, maternal baseline information (including age, BMI, gestational age, birth order, gravidity, parity, menstrual regularity, education, urban residence, family population and income, health of other children, and Pittsburgh Sleep Quality Index (PSQI) score) was collected, and written informed consent was obtained 1 day before operation. Intraoperative information (total time, anesthesia time, surgery time, lumbar puncture clearance, level of anesthesia, drugs for lumbar anesthesia, VAS for visceral traction pain during operation, fluid intake, bleeding volume, urine volume, 1 and 5 min Apgar score, hypotension, and/or hypertension) was collected on the day of surgery.

#### Outcome Indicator Collection

Before entering the operating room, parturients were arranged to the preoperative preparation room for checking parturient information, peripheral venous cannulation and so on, at which they were evaluated with the PRAQ-R2 and EPDS scales (T0). After the anesthesia was administered, when the maternal vital signs were stable, and before surgery begins, the study staff again assessed the PRAQ-R2 and EPDS scales, which would be completed within 5–10 min (T1). After cesarean section, parturients were evaluated before leaving the operating room, and we evaluate their PRAQ-R2 and EPDS for the third time (T2). At 24 h after cesarean section, parturients were evaluated in the maternity ward, including PRAQ-R2 and EPDS, postpartum childbearing fatigue, maternal satisfaction, VAS after operation, and postoperative complications such as nausea and vomiting, chill, dyspnea, and dizziness (T3).

#### Measures

##### Pregnancy-Related Anxiety Questionnaire-Revised 2

Pregnancy-Related Anxiety Questionnaire-Revised 2 (34) was the primary outcome measure used to measure anxiety level. The PRAQ-R2 contains 10 items with its response score ranging from 1 to 5, with higher scores indicating higher levels of pregnancy-related anxiety. Primiparous women with PRAQ-R scores of ≥26 and parous women with PRAQ-R scores of ≥21 are considered to be suffering from anxiety (35).

##### Edinburgh Postnatal Depression Scale

Edinburgh Postnatal Depression Scale (36) is a self-report questionnaire consisting of 10 items, with a 4-point Likert scale (0–3), designed to assess postpartum depression. This scale addresses the intensity of depressive symptoms within the previous 7 days and has been used in several studies both with pregnant and postpartum women, namely, in Portugal (37). The threshold for postpartum depression was defined as a total score of ≥13. The higher the score, the more severe the depressive symptoms.

##### Maternal Satisfaction Scale for Cesarean Section

This questionnaire consists of 22 items specifically designed to assess maternal satisfaction with neuraxial anesthesia for elective cesarean section. Satisfaction with four elements is assessed: the anesthetics, insertion of the needle into the back, the side effects, and the atmosphere in the theater. Each item is scored on a 7-point scale, and the scores are added to give a total score (minimum score 22 and maximum score 154), with a higher score representing higher satisfaction (38).

##### Clinical Approaches in the Assessment of Postpartum Fatigue

In the Fatigue Identification Form, the mother chooses from several symptom-related adjectives. Scores range from a low of 30 to a high of 120 (39).

##### Visual Analogue Scale

A 100 mm VAS is by far the most frequently used assessment instrument to evaluate analgesic effects of various therapies and detect minute pain changes during analgesic administration. Participants were asked to make a hatch mark on a 100 mm line that represents their average pain intensity: 0–3 points for mild pain, 4–6 points for moderate pain, and 7–10 points for severe pain (40).

##### Pittsburgh Sleep Quality Index

PSQI is a widely used and well-validated 19-item self-administered survey designed for the subjective evaluation of sleep quality and disturbances in clinical populations. It provides a global score ranging from no sleep difficulty to severe difficulties. The 19 items are categorized into seven clinically derived components including sleep duration, disturbances during sleep, sleep latency, dysfunction during the day due to sleepiness, efficiency of sleep, overall sleep quality, and need medication to sleep. Each component score is weighted equally from 0 to 3, and PSQI score is calculated by adding the scores for each question to obtain a global score (0–21). Higher global PSQI scores indicate poorer sleep. PSQI was used to understand maternal postnatal sleep (41).

Additional outcomes included nausea and vomiting, chill, dyspnea, and dizziness.

### Statistical Analysis

#### General Considerations

The sample size estimate of this study was predetermined and posted on a publicly accessible server.^2^ The principle of data analysis and the statistical plan were decided before the initiation of the study. This experiment used the principle of intentionality analysis and interpolated the data using the random forest method.

#### Sample Size Calculation

The sample size was determined *a priori* using PASS15. Our preliminary data showed that after stimulation, the mean anxiety score of the intervention group was 14.65, and the standard deviation was 3.82, while that of the control group was 16.83. We chose a study power of 0.80 and a significance level of 0.05 and used a two-sided significance level to find significant difference in mean scores; we then derived that 49 patients per group were required. Considering 20% loss of follow-up, the sample size was increased to 63 per group. Thus, 126 patients were recruited and randomized into two groups. All subjects completed the intervention period and follow-up, and there were no dropouts.

#### Statistical Procedures

Normally distributed data are represented by mean ± SD. Non-normally distributed data are represented by median (interquartile range). Count data are expressed by number (percentage). The Shapiro–Wilk and Levine’s test were applied to assess the normality of the distribution and homogeneity of variance of the data, respectively. The repeated measurement data, such as anxiety score and depression score at various time points of T0, T1, T2, and T3, were compared using a linear mixed-effects model. The linear mixed-effects model was performed using the lmerTest package in the R software (R version 3.6.1). The group, time (modeled as a categorical variable), and group-and-time interaction were fixed effects, and the random effect was a random intercept for subjects. Secondary comparisons were made using *t*-test for parametric, continuous data; Mann–Whitney U test for non-parametric, continuous data; and Fisher’s exact tests for binomial data. Finally, dividing them into anxiety and non-anxiety groups based on the anxiety score before leaving the operation room (T2). The glm function in R software is used to perform a single-factor logistic regression analysis to explore the risk factors that may affect anxiety. Then the variables with *P* < 0.1 in the single-factor logistic regression analysis are included in the multi-factor logistic regression analysis. *P* < 0.05 was considered statistically significant, and the R software for Windows was used for all statistical analyses.

## Results

### Demographic and Clinical Characteristics

**FIGURE 1:**
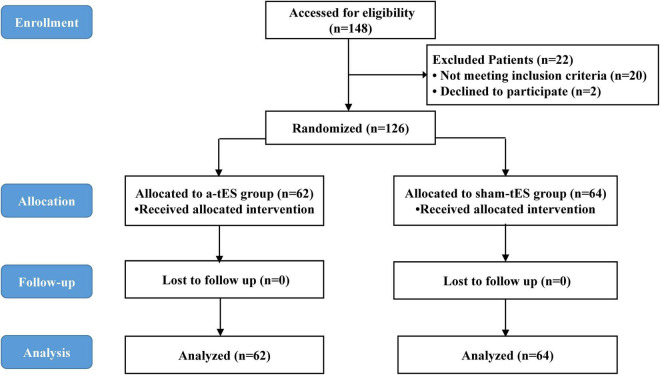
CONSORT flow diagram.

**FIGURE 2:**
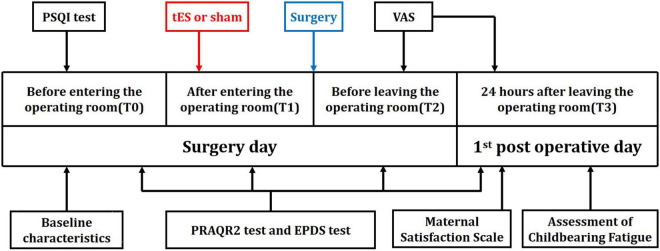
Timeline of experimental design. Experimental design and timeline of the two experimental sessions (active-tES and sham-tES).

One hundred and forty-eight parturients presenting for elective cesarean section were approached for participation in the study between March 2021 and May 2021. Among them, 126 eligible parturients were enrolled in the a-tES group (N = 62) and sham-tES group (N = 64). During the study period, no complications were observed among patients who completed the treatment protocols and who tolerated the tES treatments well. Data from all parturients were analyzed according to their assigned group. The flow chart of the study with parturient enrollment, allocation, follow-up, and analysis is shown in Figure 1. Experimental design and timeline of the two experimental sessions (active-tES and sham-tES) are shown in Figure 2. There were no significant differences in sociodemographic and clinical variables at baseline between the groups (Table 1).

**TABLE 1:** Sociodemographic and clinical variables at baseline.

Variable	sham-tES (n = 64)	a-tES (n = 62)	χ^2^/*Z*/*t*	*P*-Value
Age (years)	30.39 ± 5.14	30.82 ± 4.24	-0.51	0.608
Height (cm)	162.95 ± 4.95	161.31 ± 4.75	1.90	0.059
Weight (kg)	76.00 (71.50, 80.50)	75.00 (70.00, 85.00)	4.68	0.653
BMI (kg/m^2)^	28.99 (26.57, 31.63)	29.46 (26.45, 31.24)	4.94	0.941
Gestational age (days)	270.50 (264.80, 274.00)	270.50 (266.00, 273.80)	5.08	0.728
Gravidity (time)	2.00 (1.00, 3.00)	2.00 (2.00, 3.00)	4.91	0.994
Parity (time)	2.00 (1.00, 3.00)	2.00 (1.00, 2.00)	4.66	0.596
Menstrual regularity	49.00 (76.60%)	52.00 (83.90%)	0.65	0.421
**Education**				
Junior high school and below	19 (29.70%)	25.00 (29.00%)	6.02	0.331
Senior high school and above	45.00 (70.30%)	8.00 (71.00%)		
Urban residence	29.00 (45.30%)	33.00 (53.20%)	0.50	0.478
Family population	3.00 (2.00, 4.00)	3.00 (2.00, 4.00)	5.36	0.346
Family income ≥10,000 (rmb)	41.00 (64.10%)	41.00 (66.10%)	1.54	0.462
Have children in poor health	1.00 (1.60%)	3.00 (4.80%)	1.17	0.603
Total time (min)	100.00 (85.00, 115.00)	100.00 (90.00, 113.75)	4.97	0.756
Anesthesia time (min)	10.00 (10.00, 12.25)	10.00 (10.00, 16.50)	4.93	0.299
Surgery time (min)	57.50 (50.00, 65.50)	55.00 (50.00, 65.00)	5.04	0.867
Lumbar puncture clearance			3.24	0.071
L2–L3	46 (71.90%)	34 (54.80%)		
L3–L4	18 (28.10%)	28 (45.20%)		
Drugs for lumbar anesthesia			1.36	0.243
Bupivacaine only	59 (92.20%)	52 (83.90%)		
Bupivacaine + Lidocaine	5 (7.80%)	10 (16.10%)		
Level of anesthesia was T6 or above	63 (98.40%)	59 (95.20%)	0.29	0.361
Fluid intake (ml)	1,250 (1,000, 1,250)	1,250 (1,000, 1,250)	4.87	0.757
Bleeding volume (ml)	400 (400, 500)	400 (400, 500)	4.97	0.847
Urine volume (ml)	200 (200, 200)	200 (200, 200)	4.35	0.709
1 min Apgar	9.00 (9.00, 9.00)	9.00 (9.00, 9.00)	5.07	0.623
5 min Apgar	10.00 (10.00, 10.00)	10.00 (10.00, 10.00)	5.15	0.776
Hypotension	12 (18.80%)	14 (22.60%)	0.10	0.756
Hypertension	5 (7.80%)	13 (21.00%)	0.001	0.971

*BMI, body mass index; T6, sixth thoracic vertebra.*

### Peripartum Anxiety

**FIGURE 3:**
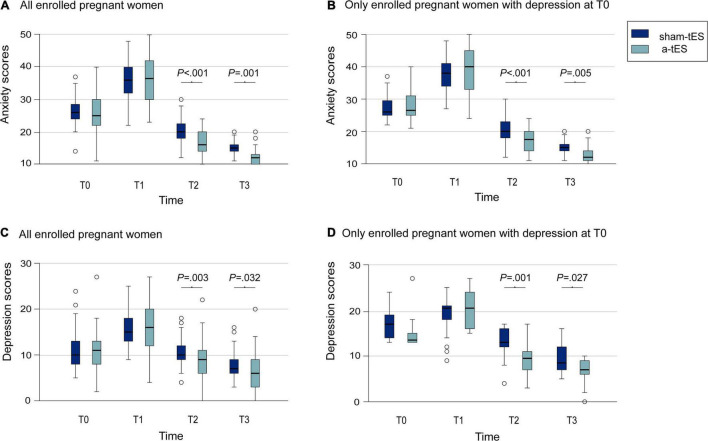
Comparison of anxiety and depression scores of pregnant women between the a-tES group and sham-tES group. **(A)** Changes in anxiety scores in all enrolled pregnant women. **(B)** Changes in anxiety scores in only enrolled pregnant women with anxiety at T0. **(C)** Changes in depression scores for all enrolled pregnant women. **(D)** Changes in depression scores for only enrolled pregnant women with depression at T0.

Throughout the trial, for all parturients, a decrease in anxiety levels was observed in the two treatment groups. Intragroup analyses showed a significant difference between T0, T1, T2, and T3. Using a mixed-effects linear model in lmerTest package of R software, we found no significant difference in scores between the two groups before stimulation, but after stimulation, anxiety scores in the a-tES group were significantly lower than that in the sham-tES group (T2: *P* < 0.001; T3: *P* = 0.001, Table 2 and Figure 3A). There were significant differences between groups and time comparisons (*P* = 0.002; *P* < 0.001, Table 2).

**TABLE 2:** Comparison of anxiety and depression between the two groups.

	T0	T1	T2	T3
**Anxiety scores of all parturients, mean (95% CI)**				
sham-tES (n = 64)	26.30 (25.30, 27.40)	36.00 (32.00, 42.00)	20.40 (19.30, 21.50)	15.00 (13.90, 16.10)
a-tES (n = 62)	25.70 (24.30, 27.10)	36.10 (34.70, 37.50)	16.10 (14.70, 17.50)	12.10 (10.70, 13.50)
*P*-values between groups	0.492	0.467	<0.001	0.001
*P*-value for time	< 0.001			
*P*-value for group and time interaction	< 0.001			
*P*-value for processing factor	0.002			
**Number and proportion of anxious mothers at each time point**				
sham-tES	55.00 (85.90%)	63.00 (98.40%)	17.00 (26.60%)	0.00
tES	42.00 (67.70%)	61.00 (98.40%)	3.00 (4.80%)	0.00
χ^2^	4.902	3.509	9.562	
*P*-value	0.477	0.492	0.002	
**Anxiety scores of parturients who were anxious at T0, mean (95% CI)**				
sham-tES (n = 50)	27.20 (26.00, 28.40)	37.70 (36.60, 38.90)	20.60 (194.0, 21.80)	15.20 (14.1, 16.4)
a-tES (n = 43)	28.60 (27.30, 30.00)	39.20 (37.90, 40.60)	17.00 (15.60, 18.30)	12.80 (11.50, 14.00)
*P*-values between groups	0.119	0.097	<0.001	0.005
*P*-value for time	<0.001			
*P*-value for group × time	<0.001			
*P*-value for processing factor	0.218			
**Depression scores of all parturients, mean (95% CI)**				
sham-tES (n = 64)	11.14 (10.22, 12.06)	15.64 (14.72, 16.56)	10.47 (9.55, 11.39)	7.64 (6.72, 8.56)
a-tES (n = 62)	10.50 (9.40, 11.60)	16.15 (15.05, 17.24)	8.27 (7.17, 9.37)	6.08 (4.98, 7.18)
*P*-values between groups	0.378	0.487	0.003	0.032
*P*-value for time	<0.001			
*P*-value for group × time	0.002			
*P*-value for processing factor	0.086			
**Number and proportion of depressed mothers at each time point**				
sham-tES	18 (28.10%)	32 (50.00%)	12 (18.80%)	5 (7.80%)
a-tES	18 (29.00%)	35 (56.40%)	6 (9.70%)	3 (4.70%)
χ^2^		0.526	1.44	0.458
*P*-value	1.000	0.470	0.230	0.490
**Depression scores of parturients who were depressed at T0, mean (95% CI)**				
sham-tES (n = 18)	16.61 (15.01, 18.22)	18.89 (17.28, 20.49)	12.89 (11.28, 14.49)	9.44 (7.84, 11.05)
a-tES (n = 18)	14.89 (13.20, 16.57)	20.44 (18.76, 22.13)	9.06 (7.37, 10.74)	6.83 (5.15, 8.52)
*P*-values between groups	0.143	0.186	0.001	0.027
*P*-value for time	< 0.001			
*P*-value for group and time interaction	0.002			
*P*-value for processing factor	0.029			

*P-value for group × time, P-value for group-and-time interaction.*

Then we compared the changes in the number and proportion of anxious mothers at each time point and found that the a-tES group was significantly lower than the sham-tES group at T2 (*P* = 0.002, Table 2). But the number of both groups was 0 at T3; this requires us to carry out further experiments to verify and explore.

Further sensitivity analysis showed that for parturients who were anxious at T0, we also found that the anxiety scores after stimulation was significantly lower than those in the control group (T2: *P* < 0.001; T3: *P* = 0.005, Table 2 and Figure 3B), consistently with before. Time differences in anxiety scores were also statistically significant (*P* < 0.001).

### Peripartum Depressive Symptoms

Meanwhile, we find that depression scores of all parturients were lower in the a-tES group after stimulation (T2: *P* = 0.003; T3: *P* = 0.032; Table 2 and Figure 3C). Time differences were equally significant (*P* < 0.001).

There were no significant differences in the change in numbers and proportion of mothers who have depressive symptoms (T2: *P* = 0.23, T3: *P* = 0.49, Table 2).

Exploring even further, for parturients who have depressive symptoms at T0, we carried out sensitivity analysis and also found lower depression scores in the a-tES group after stimulation (T2: *P* = 0.001; T3: *P* = 0.027; Table 2 and Figure 3D). The difference in scores between the two groups changed significantly over time (*P* < 0.001).

### Comparison of Other Outcomes

tES had no effect on intraoperative nausea and vomiting, chills, and dyspnea but reduced the incidence of dizziness in the intervention group (*P* = 0.018) (Table 3). VAS score during operation was the maximum value of visceral traction pain from delivery of the fetus to completion of suturing. VAS scores during and after operation were lower in the tES group (*P* = 0.040; *P* = 0.012, Table 3). There was no difference in maternal satisfaction score, postpartum fatigue scores score, and PSQI score between groups (Table 3).

**TABLE 3:** Comparison of other outcomes between the two groups.

Variables	sham-tES (n = 64)	a-tES (n = 62)	χ ^2^/*Z*/*t*	*P*-value
Nausea and vomiting	59 (92.20%)	49 (79.00%)	3.44	0.065
Chill	2 (3.20%)	3 (4.80%)	0.001	0.675
Dyspnea	3 (4.70%)	7 (11.50%)	1.08	0.200
Dizziness	1 (1.60%)	9 (14.50%)	5.56	0.018
VAS during operation	2.50 (2.00, 3.00)	2.00 (0.00, 3.00)	3.96	0.040
VAS after operation	6.02 (4.00, 7.05)	4.01 (3.00, 6.00)	3.53	0.012
Satisfaction score	118.50 (116.00, 124.50)	119.50 (111.00, 122.50)	4.41	0.322
Fatigue score	70.00 (65.00, 73.00)	67.50 (65.00, 72.00)	4.11	0.107
PSQI score	9.00 (7.00, 11.00)	8.00 (6.25, 10.00)	4.35	0.262

*VAS during operation: The maximum value for visceral traction pain from delivery of the fetus to completion of suturing; PSQI: Pittsburgh Sleep Quality Index.*

### Logistic Regression

Specifically, according to parity, patients with an anxiety score less than 21 points were evaluated as non-anxious, and patients with a score greater than or equal to 21 points were evaluated as anxious. For patients with a parity more than 1, those with an anxiety score of less than 26 were mediated as non-anxious, and those with an anxiety score greater than or equal to 26 were considered to have anxiety. A two-category logistic regression analysis was carried out to determine whether there is anxiety as a two-category outcome indicator.

Single factor logistic regression analysis shows that the *P*-values of groups gravidity, parity (time), occupation, education, and lumbar puncture interspace are less than 0.1 (Table 4). Incorporating these factors into the multivariate logistic regression analysis, we found that only the group showed significant statistical differences. There was no difference in other indicators, further indicating that the two groups were balanced and comparable, and the intervention group was less likely to have anxiety than the control group (Table 5).

**TABLE 4:** Univariate logistic regression.

Variable	OR	95% CI	*P*-value
Age (year)	0.97	(0.88, 1.07)	0.565
Height (m)	0.96	(0.88, 1.05)	0.397
Weight (kg)	0.99	(0.95, 1.03)	0.717
BMI (kg/m^2^)	1.00	(0.89, 1.12)	0.959
Gestational age (day)	0.95	(0.88, 1.01)	0.121
Gravidity (time)	1.36	(1.05, 1.80)	0.024
Parity (time)	2.79	(1.53, 5.44)	0.001
Menstrual regularity	0.73	(0.27, 2.23)	0.561
**Education**			
Junior high school and below	Reference	Reference	1.000
Senior high school	0.74	(0.20, 2.44)	0.628
High school	0.36	(0.13, 0.97)	0.044
Occupation	0.34	(0.11, 0.87)	0.032
Urban residence	0.51	(0.20, 1.24)	0.144
Family population	1.25	(0.90, 1.75)	0.172
**Family income (rmb)**			
<10,000	Reference	Reference	1.000
≥10,000	0.62	(0.25, 1.53)	0.290
PSQI score	1.09	(0.93, 1.29)	0.292
Fatigue score	1.02	(0.99, 1.08)	0.251
Satisfaction score	1.03	(0.98, 1.09)	0.221
VAS during operation	1.10	(0.85, 1.43)	0.455
VAS after operation	1.19	(0.90, 1.57)	0.240
Total time (min)	0.99	(0.95, 1.00)	0.107
Anesthesia time (min)	0.93	(0.84, 1.01)	0.121
Surgery time (min)	0.98	(0.94, 1.01)	0.245
Group	0.14	(0.03, 0.40)	0.001
Lumbar puncture clearance	2.23	(0.92, 5.49)	0.076
Drugs for lumbar anesthesia	0.59	(0.09, 2.23)	0.505
Fluid intake (ml)	1.00	(0.99, 1.00)	0.450
Hyperensort	1.03	(0.42, 2.57)	0.957
Bleeding volume (ml)	1.00	(0.99, 1.00)	0.817
Urine volume (ml)	0.99	(0.98, 1.00)	0.374
1 min Apgar	2.39	(0.90, 14.38)	0.205
5 min Apgar	2.28	(0.87, 13.53)	0.226
Hypotension	1.68	(0.59, 4.47)	0.313
Hypertension	1.01	(0.05, 7.22)	0.993
Nausea and vomiting	0.78	(0.17, 2.64)	0.716
Shivering	1.01	(0.50, 7.22)	0.993
Difficult breathing	0.42	(0.02, 2.44)	0.429
Dizzy	0.42	(0.02, 2.43)	0.429

*Reference: dummy variable, an artificial variable created to represent an attribute with two or more distinct categories/levels.*

**TABLE 5:** Multivariate logistic regression analysis.

Variable	OR	95% CI	*P*-value
Group	0.07	(0.01, 0.26)	0.000
Gravidity (time)	1.18	(0.79, 1.70)	0.366
Parity (time)	1.68	(0.69, 4.25)	0.253
Occupation	0.40	(0.11, 1.38)	0.161
**Education**			
Junior high school and below	Reference	Reference	1.000
Senior high school	0.98	(0.21,4.43)	0.977
High school	0.66	(0.17, 2.55)	0.539
Lumbar puncture	4.20	(1.31, 15.00)	0.059

*Reference: dummy variable, an artificial variable created to represent an attribute with two or more distinct categories/levels.*

## Discussion

Peripartum mental health disorders, including anxiety and depressive pregnancy, are common, and there is an increased risk of depression in pregnant women who have anxiety (42). Peripartum mental health disorder can lead to poor maternal infant physical health and negative birth outcomes. To avoid these hazards, people often use pharmacological and non-pharmacological treatments. It is well known that benzodiazepines and benzodiazepine-related drugs are generally prescribed for the treatment of anxiety disorders (43). These drugs have anxiolytic, hypnotic, and anticonvulsant properties and may relieve symptoms in the short term. There is no doubt about that benzodiazepines are effective in non-pregnant populations. However, when used during pregnancy, benzodiazepine-related drugs pass readily through the placenta, with a greater placental transfer in late pregnancy, compared to early pregnancy (44). Some work has found that neonates exposed to benzodiazepines *in utero* are more likely to have respiratory difficulties, particularly if exposure is late in gestation (45). The US Food and Drug Administration has categorized various drugs according to their risk during pregnancy and lactation (46). Most drugs, such as lorazepam, oxazepam, and diazepam, are categorized as D, indicating that there is evidence of human fetal risk (47). In view of the above considerations, we prefer to explore non-pharmacological physical therapy methods for the relief of anxiety and depression in women during pregnancy, which are non-invasive and safe and do not pose risks to the mother and fetus (48). Therefore, we discovered tES, a typical low-intensity transcranial electric stimulation, which has been proven to be effective for psychological disorders such as generalized anxiety, with a high degree of safety and almost no adverse effects on the fetus and the mother (49).

Guleyupoglu et al. reported that tES evolved from the concept of “electro-sleep” and was first investigated at the beginning of the 20th century (25). It was hypothesized that tES treatment did not in fact induce sleep, but rather the sleep was a side effect of the relaxing induced by the current stimulation, resulting in changing the name from “electro-sleep” to “transcranial electrical stimulation” (25, 50). Several studies have proposed the therapeutic efficacy of non-invasive brain stimulation for treating neurological conditions and psychiatric disorders, such as generalized anxiety disorder (GAD), depression, addiction, stroke, and pain (20, 22, 26). The authors performed the cathode positioned over the right DLPFC and the anode over the contralateral deltoid with a current of 2 mA for 30 min, and it was found that there was a significant improvement of anxiety and a discrete improvement in depression during the treatment (51).

The DLPFC has a potential role in top-down control of processes involved in mood disturbances, including the orbitofrontal cortex and medial prefrontal cortex. Since the DLPFC tonically inhibits the amygdala, neuromodulation of these nuclei could improve this inhibition (18, 52). This is essential for balancing the stress response because anxiety and depression are associated with prefrontal cortex hypoactivity and lack of inhibitory neural mechanisms (52). tES is not only employed to modulate cortical excitability in a target region but also induces changes in the interconnected areas and cortico-subcortical circuits (24, 53, 54). A meta-analysis, which included 61 single-session, sham-controlled, crossover DLPFC tDCS studies, concluded that overall participants across trials and analyses revealed a small, significant effect of a-tDCS on improving RTs and accuracy in cognitive tasks (55). They also find gender differences (i.e., stronger increase in accuracy following a-tDCS in females). Women take a more “top-down” cognitive strategy than men, relying more heavily on higher-order frontal regions, which is enhanced by DLPFC tDCS (56).

As far as we aware, this is the first randomized sham-controlled trial to assess the short-term effects of tES for peripartum mental health disorders in parturients undergoing cesarean section with combined spinal–epidural anesthesia. We found that tES performed over the DLPFC (2 mA for 20 min) ameliorated peripartum anxiety and depression score, whether for parturients who were anxious or depressed at T0, or for all mothers. There were also significant differences in the number and proportion change of anxious mothers (T2: *P* = 0.002). In the a-tES group, VAS scores during the operation and after operation were decreased (*P* = 0.040; *P* = 0.012), and the incidence of dizziness during surgery was also reduced (*P* = 0.018). However, there were no significant differences in other peripartum complications, maternal satisfaction score, postpartum fatigue score, and PSQI score between the groups.

In this trial, the intervention group outperformed the control group before leaving the operating room, both in terms of change in scores and number of anxious women only and in terms of changes in general anxiety scores. For a variety of reasons, mothers may briefly experience sudden increases in anxiety and depression levels and decreases in mental health during the perioperative period; these may be quickly relieved by the progression of surgery and anesthesia and the birth of the fetus, so that a proportion of women who are anxious before they enter the operating theater fall back to normal levels of anxiety both before they leave the theater and at the postoperative follow-up. However, it is easy to see from our data that tES did reduce maternal anxiety scores before leaving the operating theater compared to the control group scores, reducing the number and proportion of anxious people, with a statistically significant difference, and similarly, maternal depression scores were significantly reduced. More conclusions need to be verified by further research. In other words, tES improves maternal perioperative mental health. tES reduced pain scores at intraoperative and postoperative follow-up. In terms of intraoperative complications, tES did not increase the incidence of perioperative complications such as nausea and vomiting and chills in either group but reduced the incidence of dizziness, which may be related to the neurosensory effects it brings (57).

Future studies with larger sample sizes and more extended follow-up periods are needed to clarify the effectiveness of non-invasive brain stimulation for peripartum anxiety and depression that is refractory to conventional treatments. Research on its long-term effects may provide additional and more valid evidence for the role of tES in relieving peripartum mental health disorders of women undergoing cesarean section.

### Limitations

There are limitations of the pilot study that should be addressed. First, the study performed only one session of a-tES. Over the last decade, the single-session approach has been used to investigate a wide array of cognitive functions, including perception, verbal fluency, visual search, attention, etc. Despite that, studies have shown significant reductions in anxiety levels in patients with generalized anxiety disorders using multiple sessions of stimulation (58). Therefore, multiple sessions during the a-tES protocol for peripartum anxiety are recommended for future investigations to potentially increase the therapeutic efficacy. Secondly, we had a short follow-up period, and there was loss of data for long-term effects of a-tES. Thirdly, we did not follow up the newborns after the Apgar score determined in the operating room and missed the effects of a-tES on the newborns in the maternity ward. So, in a further study, we will extend the follow-up period and add more attention to the newborn.

## Conclusion

In summary, the main results of this pilot randomized study show that a-tES can improve the peripartum mental health disorders, including anxiety and depressive symptoms, of women undergoing cesarean section with combined spinal–epidural anesthesia.

## Data Availability Statement

The raw data supporting the conclusions of this article will be made available by the authors, without undue reservation.

## Ethics Statement

The studies involving human participants were reviewed and approved by the Ethical Committee of the Affiliated Hospital of Xuzhou Medical University. The patients/participants provided their written informed consent to participate in this study. Written informed consent was obtained from the individual(s) for the publication of any potentially identifiable images or data included in this article.

## Author Contributions

J-LC designed the study, gave critical review of the manuscript, approved the final version, and was accountable for the work. HL and YH designed the study, prepared the manuscript, gave critical review of the manuscript, approved the final version, and were accountable for the work. M-ST, Q-QZ, and JZ helped conduct the study and collect the data. XL, J-XF, JY, R-GL, JW, and XS analyzed and interpreted the data. QZ, X-YH, and SZ conducted the study, collected the data, prepared the manuscript, helped test samples, and analyzed the data. LZ, Mannan-Abdul, and HZ helped prepare the manuscript and gave a critical review of the manuscript. All authors contributed to the article and approved the submitted version.

## Conflict of Interest

The authors declare that the research was conducted in the absence of any commercial or financial relationships that could be construed as a potential conflict of interest.

## Publisher’s Note

All claims expressed in this article are solely those of the authors and do not necessarily represent those of their affiliated organizations, or those of the publisher, the editors and the reviewers. Any product that may be evaluated in this article, or claim that may be made by its manufacturer, is not guaranteed or endorsed by the publisher.

## Footnotes

1
http://www.chictr.org.cn/

2
www.ClinicalTrials.gov
